# Subcellular Localization of Connexin 26 in Cardiomyocytes and in Cardiomyocyte-Derived Extracellular Vesicles

**DOI:** 10.3390/molecules26216726

**Published:** 2021-11-06

**Authors:** Alessandra Falleni, Stefania Moscato, Antonietta R. M. Sabbatini, Margherita Bernardeschi, Francesco Bianchi, Antonella Cecchettini, Letizia Mattii

**Affiliations:** 1 Department of Clinical and Experimental Medicine, University of Pisa, 56126 Pisa, Italy; stefania.moscato@unipi.it (S.M.); margherita.bernardeschi@for.unipi.it (M.B.); francesco.bianchi@unipi.it (F.B.); antonella.cecchettini@unipi.it (A.C.); 2 Department of Surgical, Medical, Molecular Pathology and of Emergency Medicine, University of Pisa, 56126 Pisa, Italy; antonietta.sabbatini@unipi.it; 3 Institute of Clinical Physiology (IFC), National Research Council (CNR), 56126 Pisa, Italy

**Keywords:** connexin26, immunoelectron microscopy, H9c2 cells, rat heart, ultrastructure, cardiomyocyte, extracellular vesicles

## Abstract

Connexins (Cxs) are a family of membrane-spanning proteins, expressed in vertebrates and named according to their molecular weight. They are involved in tissue homeostasis, and they function by acting at several communication levels. Cardiac Cxs are responsible for regular heart function and, among them, Cx26 and Cx43 are widely expressed throughout the heart. Cx26 is present in vessels, as well as in cardiomyocytes, and its localization is scattered all over the cell aside from at the intercalated discs as is the case for the other cardiac Cxs. However, having been found in cardiomyocytes only recently, both its subcellular localization and its functional characterization in cardiomyocytes remain poorly understood. Therefore, in this study we aimed to obtain further data on the localization of Cx26 at the subcellular level. Our TEM immunogold analyses were performed on rat heart ventricles and differentiated H9c2 cardiac cell sections as well as on differentiated H9c2 derived extracellular vesicles. The results confirmed the absence of Cx26 at intercalated discs and showed the presence of Cx26 at the level of different subcellular compartments. The peculiar localization at the level of extracellular vesicles suggested a specific role for cardiac Cx26 in inter-cellular communication in an independent gap junction manner.

## 1. Introduction

Connexins (Cxs) are a family of membrane-spanning proteins, expressed in vertebrates and named according to their molecular weight, which can range from 26 to 60 KDa. Over twenty identified isoforms of mammalian Cxs have a shared sequence and topological homology. The latter consists of 4 transmembrane regions, 2 extracellular loops, 1 cytosolic loop, 1 cytosolic amino terminal tail and 1 cytosolic carboxy terminal tail [1]. The carboxy terminal tail and the cytoplasmic loop are the least conserved domains. Cxs are primarily involved in tissue homeostasis by acting at several communication levels. Cxs can singularly interact with regulator proteins and participate in signaling pathways that drive cell proliferation or cancer progression. Moreover, six assembled Cxs can form pores, which are named hemichannels or connexons, thereby allowing for the exchange of small molecules, such as ions, nucleotides, amino acids, sugars, mRNAs and miRNAs, with both intracellular and extracellular compartments. Finally, the connexons of adjacent cells can align to form gap junctions, allowing communication from cell to cell [1,2].

Among the cardiac Cxs, Cx26 and Cx43 are most widely expressed throughout the heart. Cx43 is expressed in blood vessels, stroma cells and working cardiomyocytes. In cardiomyocytes, they are mainly localized at the level of intercalated discs where gap junctions are formed and, continuing with its canonical function, allows for the propagation of electrical activity throughout the heart. Cx43 is also present in the mitochondria [3]. Although the functions of mitochondrial Cx43 are still poorly understood, recent studies suggested that it can affect K^+^ flux, mitochondrial Ca^2+^ homeostasis and complex I respiration and oxygen consumption [4,5,6]. Furthermore, Cx26 is present in vessels as well as in working and conducting cardiomyocytes, and its localization is scattered all over the cell but not at intercalated discs as is the case for the other cardiac Cxs [7]. The modulation of the cardiac Cx26 protein has been demonstrated both in aging [8] and in cardiac dysfunction [9]. However, having been found in cardiomyocytes only recently, its subcellular localization and its functional characterization in cardiomyocytes remain poorly understood.

Against this background, the aim of the present study is to further explore the subcellular localization of Cx26, and our results can contribute to better understand its function in cardiomyocytes. Therefore, single and double immunogold analyses were performed on rat heart samples as well as on a rat H9c2 cell line, which differentiated into a cardiac-like phenotype. Additionally, extracellular vesicles (EVs), obtained from the supernatant of differentiated H9c2 cells, were tested.

## 2. Results and Discussion

To detect the subcellular localization of Cx26 through immunogold analyses we used a rabbit anti-Cx26 previously validated on rat heart tissue [7]. The subcellular distribution of Cx26 was analysed on ultrathin sections from both rat heart tissue and cardiomyocytes obtained from the differentiation of a rat H9c2 myoblast cell line (dH9c2).

**Figure 1:**
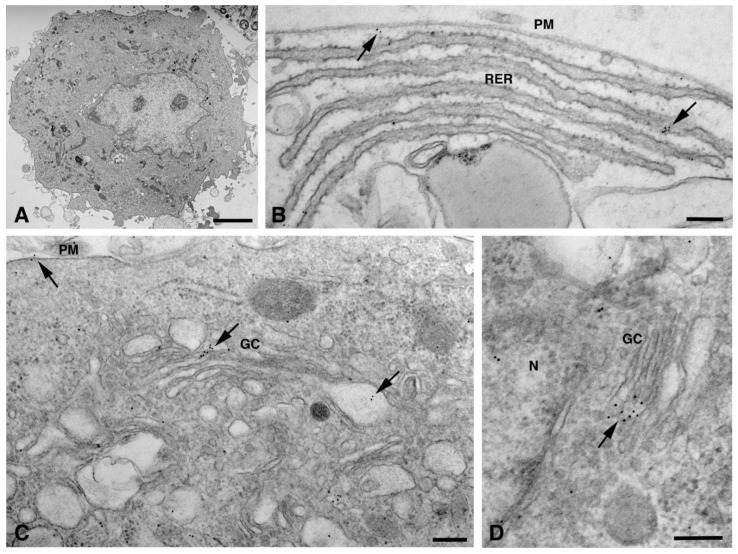
TEM immunogold analysis for Cx26 in dH9c2 cells. (**A**) Low magnification of representative cell counterstained with uranyl acetate and lead citrate without immunogold treatment. Scale bar 4 µm. (**B**–**D**) Representative images of immunoreaction (arrows) at level of different cell compartments, rough endoplasmic reticulum (RER) and Golgi complexes (GC). Scale bars 200 nm**.** N, nucleus; PM, plasma membrane.

In dH9c2 cells (representative image in Figure 1A), Cx26 was observed at the level of the endoplasmic reticulum, Golgi complex and Golgi-derived vesicles (Figure 1B–D).

**Figure 2:**
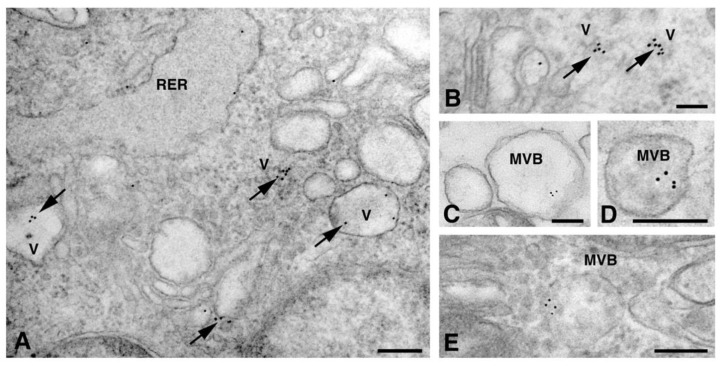
TEM immunogold analysis for Cx26 in dH9c2 cytoplasmic vesicles. (**A**,**B**) Arrows point to gold particles on different sized vesicles (V). (**C**–**E**) Representative images of multivesicular bodies (MVB) showing immunoreactivity. Scale bars 200 nm (**A**,**C**–**E**), 100 nm (**B**). RER, rough endoplasmic reticulum.

In these organelles, Cxs are respectively synthesized and oligomerized into hexamers which, being included in vesicles, are then translocated to the cell membrane or to other cell compartments [10]. However, it has been demonstrated in various tissues that Cx26, can also form oligomers spontaneously on plasma membrane, in a Golgi-independent manner, something that other Cxs cannot do [11]. Our electron micrographs show that, for the dH9c2 cytoplasm, Cx26 was found in vesicles of size ranging from 50 to 600 nm (Figure 2), including multivesicular bodies (MVBs), which are large endosomal vesicles where exosomes are formed [12].

**Figure 3:**
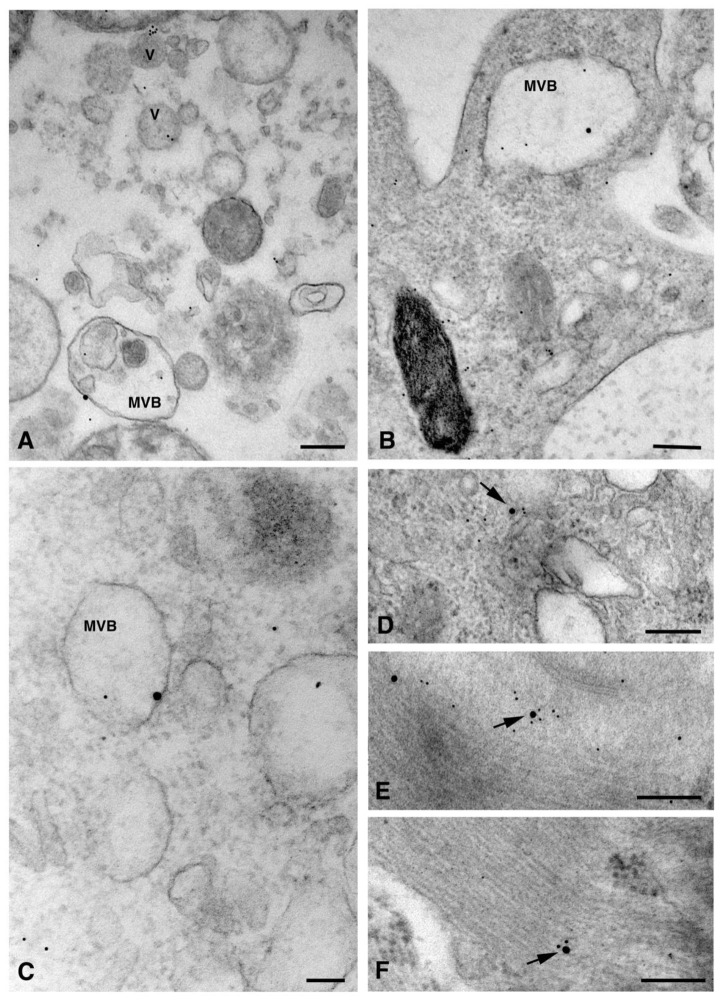
TEM double immunogold analysis for Cx26 (10 nm gold particles) and flotillin or CD63 (20 nm gold particles) on cytoplasmic vesicles. (**A**–**C**) Representative images of dH9c2 cells showing double immunoreaction for Cx26 and flotillin (**A**,**B**) and for Cx26 and CD63 (**C**) on multivesicular bodies (MVB). (**D**–**F**) Heart sections showing double immunoreactivity for Cx26 and flotillin (**D**,**E**) and for Cx26 and CD63 (**F**) on small vesicles (arrows) sometimes associated with sarcomeres (**E**,**F**). Scale bars 200 nm (**A**,**B**,**D**–**F**), 100 nm (**C**). V, vesicles.

To better understand the intracellular pathway of Cx26, we performed a double immunogold analysis by using the anti-Cx26 antibody with the antibody raised to CD63 or to flotillin, which are molecules expressed on both MVBs (100–1000 nm size) and exosomes (50–100 nm size). The results of the double immunogold analysis showed the presence of Cx26 in both MVBs and vesicles of different size (Figure 3A–C). Vesicles smaller than 100 nm, presumably exosomes, were also observed in cardiomyocytes of heart sections (Figure 3D) and, interestingly, were also observed in the sarcomere and its nearby regions (Figure 3E–F). As previously demonstrated by Sahoo et al. [12], free exosomes are found in the intracellular space embedded among the sarcomeres, nucleus, and T-tubule, but not enclosed within a multivesicular endosome-like structure.

**Figure 4:**
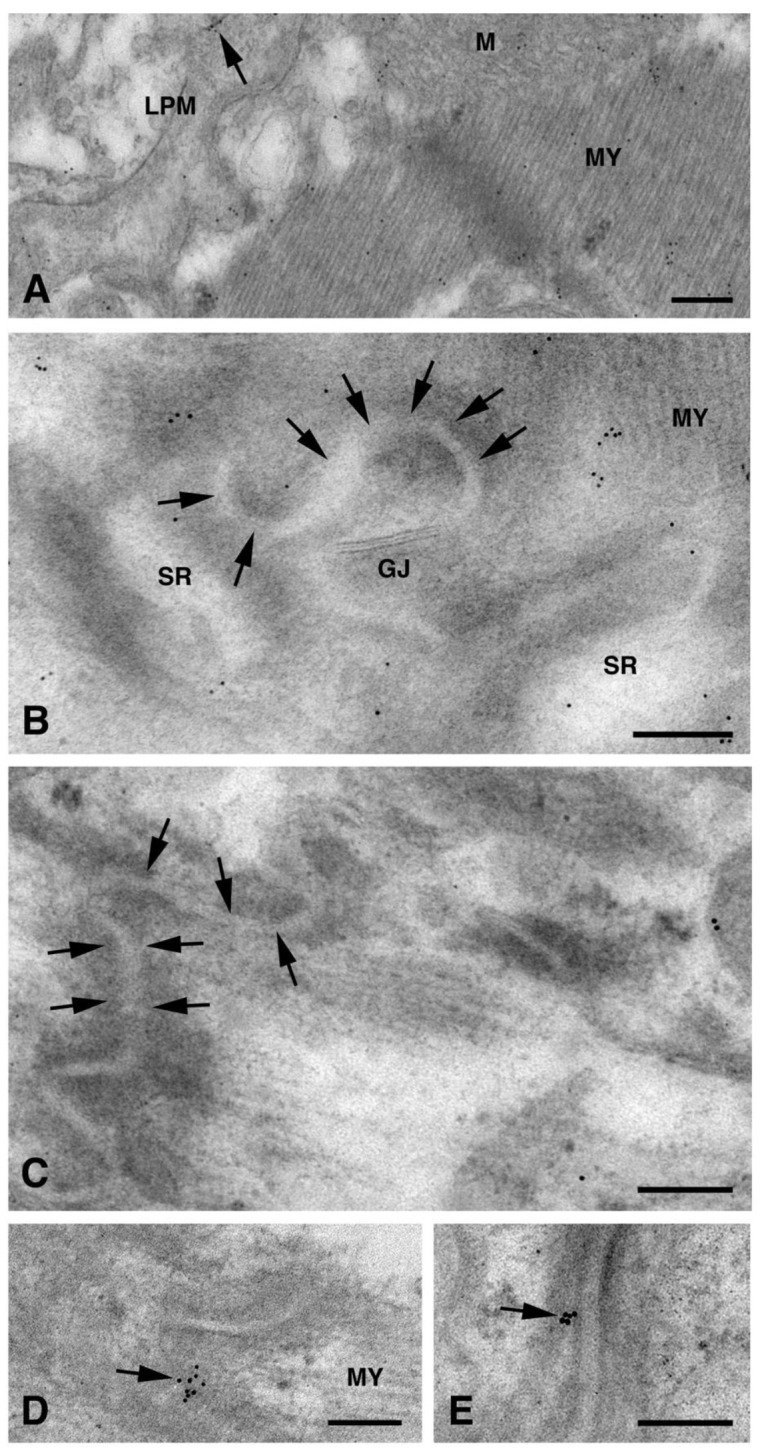
TEM immunogold analysis for Cx26 and Cx43 in cardiomyocytes of heart tissue. (**A**–**C**) Cx26 immunogold analysis. (**A**) Lateral plasma membrane (LPM) of two adjacent cardiomyocytes showing scarce amount of gold particles (arrow). Scale bar 200 nm. (**B**,**C**) Representative images showing no immunoreaction on intercalated discs (arrows) as well as on gap junctions (GJ). Scale bars 100 nm. (**D**,**E**) Cx43 immunogold analysis. Arrows point to gold particles mainly at level of intercalated discs. Scale bars 200 nm. M, mitochondrion; MY, myofibrils; SR sarcoplasmic reticulum.

Moreover, a limited level of Cx26 plasma membrane localization was present at this point on the dH9c2 cells (Figure 1B–C). On the cardiomyocytes of heart tissue, a scarce immunoreaction was found at the level of the lateral plasma membrane (Figure 4A) while immunogold staining was not present at the level of the longitudinal ends where intercalated discs and gap junctions are represented (Figure 4B–C). This is of interest because the other cardiac Cxs are primarily observed on these subcellular heart structures. We also wanted to confirm this last statement in our heart samples and thus we performed the immunogold analysis using an anti Cx43 primary antibody. As expected, the results revealed the presence of Cx43 mainly at the level of the intercalated discs [Figure 4D–E].

**Figure 5:**
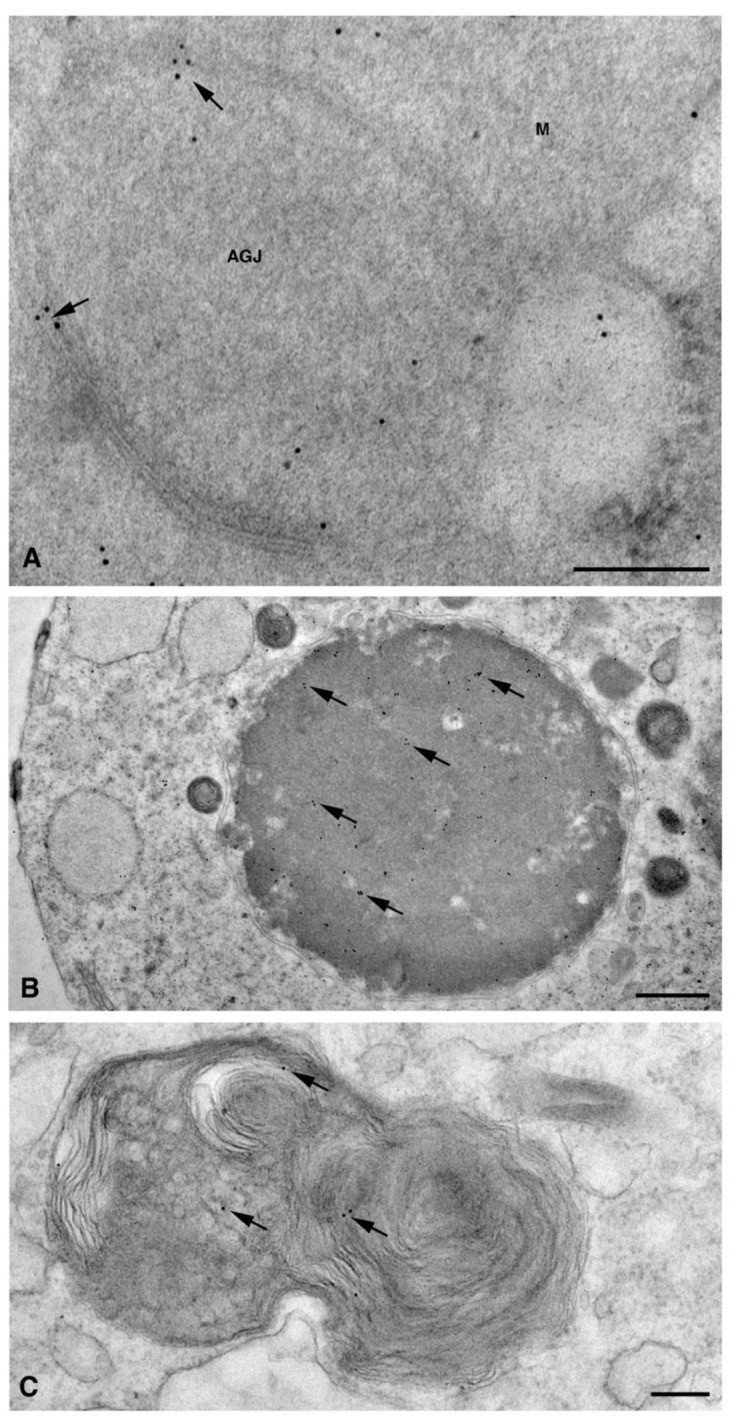
TEM immunogold analysis for Cx26 in delivery and degradation of subcellular structures. (**A**) Cardiomyocytes of heart tissue: representative image of an annular gap junction (AGJ), associated with a mitochondrion (M), showing immunogold reaction on (arrows) and inside the membrane. Scale bar 200 nm. (**B**,**C**) Representative images of autophagic vacuoles showing scattered gold particles (arrows) in dH9c2 cells. Scale bars 600 nm (**B**), 200 nm (**C**).

Interestingly, in the cardiomyocytes of the heart tissue, Cx26 was found in double-membrane vesicles, which are occasionally associated with mitochondria (Figure 5A). These double membrane-vesicles, that presumably represent the annular gap junctions, also named connexosomes, have been demonstrated to derive from the internalization of plasma membrane gap junctions at the onset of the Cxs degradation process, which involves endolysosome or autophagosome pathways [13]. Our data suggested that the annular gap junctions could represent a Cx26 delivery system to reach other organelles such as mitochondria, as has already been demonstrated for Cx43 [14]. Moreover, the involvement of Cx26 in the intercellular organelle transfer cannot be ruled out. Indeed, a recent study [15] demonstrated that connexosome, derived from a Cx43 gap junction internalization, can incorporate mitochondria and endosomes, transferring them into a contacting cell. As a consequence, the connexosome-Cx43 internalization process could potentially facilitate both the transfer of organelles and the release of molecules from these enclosed organelles into the cytosol of the receiving cell. Finally, the detection of Cx26 in autophagic vacuoles of dH9c2 (Figure 5 B–C) revealed a degradation process similar to other Cxs, at least for final steps.

**Figure 6:**
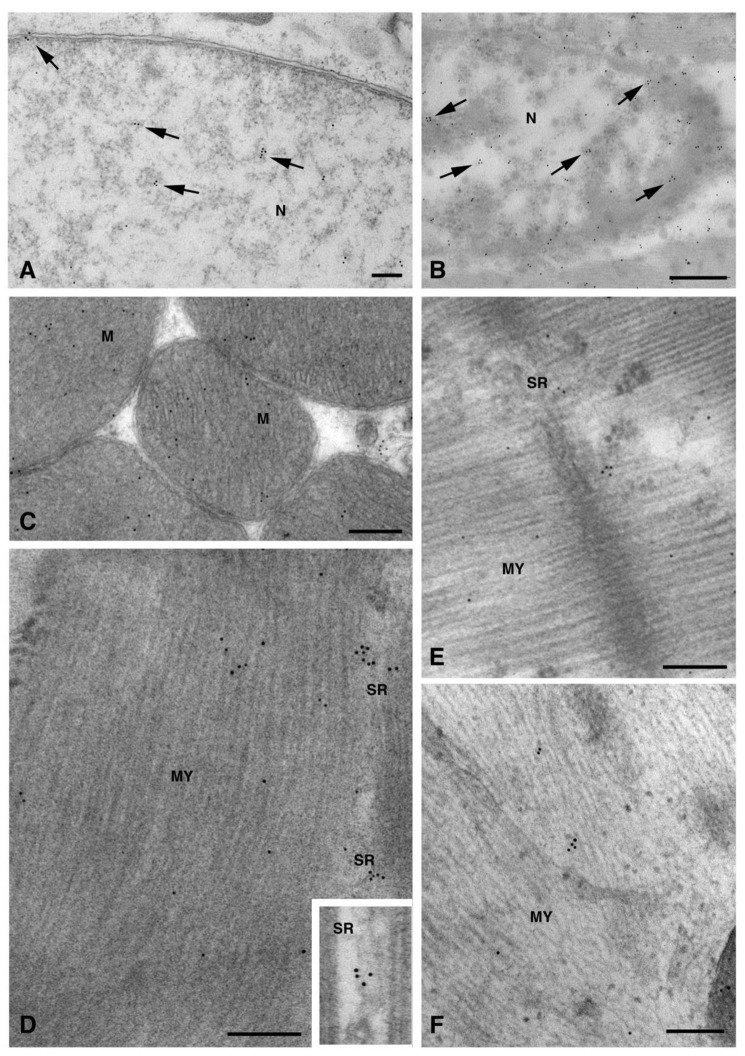
TEM immunogold analysis for Cx26 in non-canonical subcellular structures. Representative images of immunopositivity (arrows) in nucleus (N) of dH9c2 cells (**A**) and heart tissue cardiomyocytes (**B**). (**C**–**E**) Representative images of heart tissue cardiomyocytes evidencing immunogold particles on the inner membrane and cristae of mitochondria (M), on myofibrils (MY) and sarcoplasmic reticulum (SR). (**F**) Representative images of immunogold particles on myofibrils (MY) of dH9c2 cells. Scale bars 200 nm (**A**,**C–F**), 500 nm (**B**).

The presence of Cx26 on non-canonical cell structures including nuclei, mitochondria, myofibrils, and sarcoplasmic reticulum (SR) has been found, for the first time, in the present study. In particular, mitochondrial immunopositivity was localized on both the inner membrane and, in a larger amount, on the cristae (Figure 6).

These non-canonical Cxs localizations were previously found with regard to Cx43. The presence of nuclear Cx43 either as a full-length protein or as N-terminally truncated fragments has been demonstrated in tumoral cells and in cardiomyocytes. Although the nuclear Cxs functions are still unknown, their involvement in the modulation of cell growth and differentiation through the regulation of transcription at the cell nucleus have been proposed [16]. A similar role cannot be discounted for Cx26, as a previous study on human breast tumor cells demonstrated the ability of Cx26 to regulate the expression of some genes related to angiogenesis, including in a gap junction-independent manner [17]. Cxs mitochondrial localization was described for Cx43 [3,4,5,6] and for Cx40 [18]. Among the functions attributed to mitochondrial Cx43, the involvement in mitochondrial Ca^2+^ homeostasis has also been suggested for Cx40. Thus, a similar function could be ascribed to mitochondrial Cx26 too. We observed Cx26 at the level of SR (Figure 6D–E). So far, only Cx43 has been found to be associated with cardiac SR dyads at the intercalated discs, where its hemichannels are activated by a subsarcolemmal increase in Ca^2+^ during caffeine-induced SR Ca^2+^ discharge [19].

The cardiac tissue is known to actively secrete EVs, which provide the long-distance delivery of complex messages [20]. As stated in the MISEV 2018 guidelines [21], EV is the generic term for particles of different sizes, naturally released from cells and delimited by membranes. EVs can transfer their cargo by fusing with plasma membrane or via cell internalization. Moreover, it has been demonstrated that Cx43, embedded in an exosomal membrane in the form of a hexameric channel, mediates the transfer of molecules between exosomes and cells [22]. Cx43 was found at the membrane of the exosomes isolated from cell culture supernatants of H9c2 and HL-1 cardiac cell lines [22]. Interestingly, several types of cellular stress can induce an increase of exosome release from cell. In a recent study dealing with traumatic brain injury, the responsibility of the enhanced exosome release was ascribed to Cx43 [23]. Based on these factors, we wanted to explore the potential presence of Cx26 in EVs released by dH9c2.

**Figure 7:**
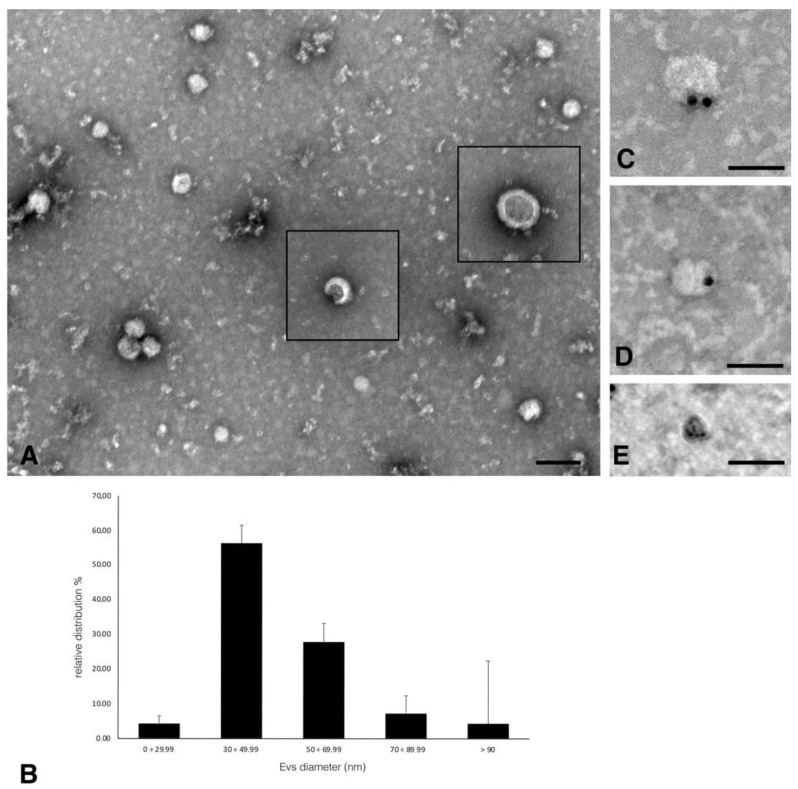
Negative staining of EVs from dH9c2 supernatant and Cx26 immunolocalization. (**A**) Representative images of EVs having a roundish and cup-shaped (squares) morphology. Scale bar 100 nm. (**B**) Diagram showing the EVs relative distribution in different dimension classes. The total number of counted EVs was 429. (**C**–**E**) Representative images of Cx26 immunogold localization performed with 20 nm (**C**,**D**) and 10 nm (**E**) gold particles conjugated anti rabbit secondary antibodies. Scale bars 100 nm.

EVs collected from a cell culture medium by ultracentrifugation were negatively stained and analyzed by TEM (Figure 7A). We observed different size EVs, some of which showed the typical collapsed cup-shaped morphology (squares in Figure 7A) due to the technical procedure of sample drying. The relative distribution of EVs in different dimension classes, reported in Figure 7B diagram, indicated that 55% of EVs belong to the 30–49.99 nm class, 30% belong to the wider 50–69.99 nm class while the other classes were less represented (<10%)., We performed an immunogold analysis for Cx26 on isolated EVs and the results showed the presence of Cx26 on the membrane of the EVs with a size ranging between 50 and 100 nm (Figure 7C–E).

This study demonstrated the presence of Cx26 in the membrane of EVs derived from cardiomyocytes for the first time. Consequently, Cx26 could form a hemichannel at the EV membrane that mediates the transfer of cargo into target cells, as was previously demonstrated for exosomal Cx43 [22]. On the other hand, EV Cx26 could contribute, in a channel-independent manner, to the recruitment and transfer of genetic information since several RNA- and DNA-binding motifs have been predicted in the Cx26 sequence [2]. The experimental results reported in this work on the subcellular and EVs localization of cardiac Cx26, represent a useful starting point for further studies aimed at investigating the functions of cardiac Cx26.

## 3. Materials and Methods

### 3.1. Rat Sample Collection

The experimental procedures were approved by the ethical committee of the University of Pisa (protocol no. 51814/2016). The investigation conforms with the Guide for the Care and Use of Laboratory Animals published by the US National Institutes of Health (NIH publication no. 85–23, revised 1996) and with the National (DL 26/2014) and European (2010/63/UE) guidelines for handling and use of experimental animals.

Heart tissue samples were collected from 12 week-old male Wistar rats (*n* = 2) (Charles River, Calco, LC, Italy). Rats were euthanized using a lethal dose of chloral hydrated (Sigma-Aldrich, St Louis, MO, USA) and their hearts were dissected. After removal, the hearts were immediately trimmed into small blocks (1 mm^3^), the ventricle samples were fixed in 1% (*w*/*v*) glutaraldehyde-4% (*w*/*v*) formaldehyde (freshly obtained from paraformaldehyde) in phosphate-buffered saline (PBS 0.1 M, pH 7.2) for 4 h at 4 °C and, after washing them in the same buffer, the specimens were postfixed in 1% (*w*/*v*) OsO_4_/PBS for 2 h. This method, which combines aldehyde and mild OsO_4_, allows for a minimal cover of antigen epitopes while preserving cell architecture and sub-cellular structures [24].

Samples of myocardium were then washed in distilled water, dehydrated in a graded series of ethanol and transferred to a propylene oxide for 6 min. Finally, embedding, using Poly/Bed 812, in a flat mold at 60 °C for 48 h, was carried out, and ultrathin sections were cut for a transmission electron microscopy (TEM) analysis.

### 3.2. Cell Culture and Extracellular Vesicles Harvesting

H9c2 rat cells were grown in Dulbecco’s modified Eagle’s Medium high glucose (DMEM) using a 10% fetal bovine serum (FBS) and antibiotics (25 U/mL penicillin and streptomycin) (all from Sigma-Aldrich) in 6 well plates and maintained at 37 °C, in a 5% CO_2_ humidified atmosphere. The differentiation of H9c2 cells into cardiomyocyte phenotype was performed as previously described [7]. In brief, cells were cultured in DMEM containing 1%FBS, antibiotics and 50 µm retinoic acid (RA), for 10 days. An FBS deprived of EVs by ultracentrifugation at 100,000× *g* for 2 h was used in the last medium change. Then, the cell culture medium was harvested, centrifuged at 300 *g* to remove cell debris and further centrifuged at 2000× *g* for 30 min at 4 °C to remove any apoptotic bodies. The supernatant underwent ultracentrifugation at 100,000× *g* for 2 h at 4 °C in order to obtain EVs. A pellet containing EVs was fixed in 2% paraformaldehyde (PFA) diluted in a 0.1 M cacodylate buffer and processed for negative staining in TEM and immunoelectron microscopy.

Differentiated H9c2 (d-H9c2) cells were detached in D-PBS using a scraper, centrifuged and fixed in 4% PFA and 0.1% glutaraldehyde in a 0.1M cacodylate buffer for 2 h and postfixed in 1% (*w*/*v*) OsO_4,_ in the same buffer, for 1 h at room temperature. Cells were then dehydrated in a graduated series of ethanol, embedded in Epon-Araldite, polymerized at 60 °C for 72 h and finally processed for the immunoelectron microscopy analysis.

### 3.3. Immunoelectron Microscopy

#### 3.3.1. Hearts and d-H9c2 Cells Post-Embedding Technique

Ultrathin sections (60–80 nm) were obtained from rat myocardium and a d-H9c2 pellet with a Reichert-Jung Ultracut E equipped with a diamond knife and collected on 200-mesh formvar/carbon coated nichel grids. The grids were incubated with a NaIO_4_ saturated aqueous solution for 30 min at room temperature to partially remove OsO_4_ and unmask antigens [25,26]. To block non-specific antigenic sites, nickel grids were incubated in a cold PBS-blocking solution containing 10% normal goat serum and 0.2% saponin for 20 min. The grids were then incubated overnight in a humidified chamber at 4 °C, with a single primary antibody, RαCx26 (diluted 1:50 in 1% goat serum/0.2% saponin/PBS; NBP2-41304 Novus Biologicals, CO, USA) or, in order to detect a co-localization, with two primary antibodies, RαCx26 and Mαflotillin or RαCx26 and MαCD63 (both diluted 1:50 in 1% goat serum/0.2% saponin/PBS; sc-28320, Santa Cruz Biotechnology, Texas, USA; MAB15170, Abnova, Taipei, Taiwan). Some of the ultrathin sections of rat myocardium were incubated with RαCx43 primary antibody (diluted 1:50 in 1% goat serum/0.2% saponin/PBS; 71-0700, Thermo Fisher Scientific, MA, USA). After rinsing in cold PBS, the grids were incubated with 10 nm gold-conjugated anti rabbit and/or 20 nm gold-conjugated anti mouse secondary antibodies (diluted 1:20 in in 1% goat serum/0.2% saponin/PBS; AC-10-01-05, Cytodiagnostics, ON, Canada; ab27242, Abcam, Cambridge, UK) for 1h at room temperature. After washings in PBS, ultrathin sections were treated with 1% glutaraldehyde for 3 min, washed in distilled water to remove salt traces and counterstained with uranyl acetate and lead citrate.

**Figure 8:**
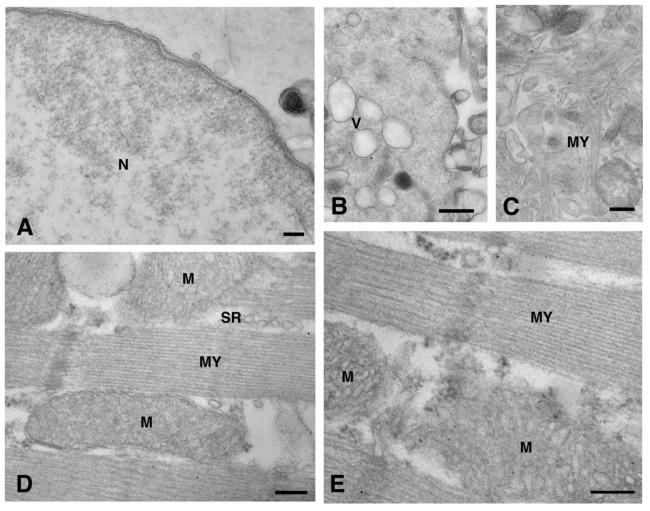
Negative controls for TEM immunogold analysis. Negative controls were obtained by omitting the primary antibody (**A**–**D**) or by using RαCx32 as primary antibody (**E**). Representative images of dH9c2 cells (**A**–**C**) and cardiomyocyte sarcoplasm of heart sections (**D**,**E**) showing no immunoreaction. Scale bars 200 nm (**A**,**C**–**E**), 500 nm (**B**). M, mitochondrion; MY, myofibrils; N, nucleus; SR, sarcoplasmic reticulum; V, vesicle.

The negative controls included (1) omitting the primary antibody and incubating the sections with the secondary antibody only for the specificity of the secondary antibody, and (2) using a rabbit IgG antibody raised to Cx32 (RαCx32, diluted 1:50 in 1% goat serum/0.2% saponin/PBS; 71-0600, Thermo Fisher Scientific) as the primary antibody for the specificity of the primary antibody (Figure 8). Similarly, we previously demonstrated, using an immunoperoxidase analysis, that RαCx32 had not reacted with heart tissue [7].

Ultrathin sections were observed using a Jeol 100SX (Japan) transmission electron microscope operating at 80 kV. Micrographs were obtained with an AMTXR80b Camera System.

#### 3.3.2. Extracellular Vesicles Negative Staining

The EVs were treated according to the method of Soares et al. [22]. To summarise the process briefly, 10 µL of the sample containing EVs fixed in 2% paraformaldehyde in 0.1 M cacodylate buffer were placed onto 200-mesh formvar/carbon-coated nickel grids which were then allowed to settle for 3 min at room temperature. After washing in PBS, the grids were treated with 50 µL of glycine for 10 min in order to block the free aldehyde groups and were then treated with the blocking buffer (PBS/0.1% BSA) for 30 min. Then grids were incubated with the primary antibody, RαCx26 (diluted 1:50 in PBS/0.1%BSA) overnight in a humidified chamber at 4 °C. After several washings in PBS, the grids were incubated in 10 or 20 nm gold-conjugated anti-rabbit secondary antibodies (diluted 1:20 in PBS/0.1%BSA) for 1h at room temperature. After rinsing in dH_2_O, the grids were finally processed for negative staining. In brief, a 20 µL aqueous solution of uranyl acetate (2% *w*/*v*) was dropped onto a grid as a staining solution. The excess was removed with filter paper after 30 s., The uranyl acetate solution was filtered through 0.45 μm polycarbonate filters to avoid any impurities before their deposition. Finally, the grids were air-dried for around 15 min and observed using a Jeol 100SX transmission electron microscope operating at 80 kV.

Micrographs at 20,000–40,000× direct magnification were obtained with an ATMxR80b Camera System. The EVs diameter was determined using the ImageJ program.

## 4. Conclusions

We demonstrated that the cardiomyocyte subcellular distribution of Cx26 differs from that of other cardiac Cxs mainly due to its scarce presence at the level of the plasma membrane and its absence at intercalated discs and gap junctions. By contrast, Cx26 was found at the level of non-canonical cell structures, including at the nucleus, mitochondria, and SR. Interestingly, it was present on the membrane of cytoplasmic vesicles and extracellular vesicles from cardiomyocytes, suggesting that cardiac Cx26 could mainly be involved in gap junction-independent, intra- and inter-cellular communication.

## Data Availability

Not applicable.
